# A Medley of Malnutrition and Myotonic Dystrophy: Twice Unlucky

**DOI:** 10.7759/cureus.21180

**Published:** 2022-01-12

**Authors:** Hajira Z Malik, Gaurav Sharma, Cesar Moreno, Siva P Parcha

**Affiliations:** 1 Internal Medicine, University of South Alabama, Mobile, USA

**Keywords:** thiamine deficiency, progressive dysphagia, steinert’s disease, wernicke encephalopathy, hyporeflexia, myotonic dystrophic type 1

## Abstract

Micronutrient and vitamin deficiencies in young adults in the United States are relatively rare and often pose a diagnostic challenge. Here, we present the case of a young female who developed acute encephalopathy and muscular weakness two days following an endoscopic procedure performed to investigate the patient’s four-month history of intractable nausea, vomiting, dysphagia, and weight loss. She was diagnosed with severe Wernicke encephalopathy due to thiamine deficiency as well as myotonic dystrophy type 1 (DM1). The patient’s family history revealed an undiagnosed muscular disorder that required her father to ambulate with a wheelchair in the fourth decade of his life. DM1 with 11 trinucleotide repeats of cytosine-thymine-guanine in allele 1 and more than 150 in allele 2 of the *myotonic dystrophy protein kinase* (*DMPK*) gene were found on genetic testing. The patient’s progressive dysphagia was likely a chronic manifestation of myotonic dystrophy that became more apparent following general anesthesia. DM1 is a multisystem genetic disorder of both skeletal and smooth muscles caused by deviation in the *DMPK *gene. Due to the involvement of esophageal smooth muscle, dysphagia may also be present. The long-standing dysphagia in our patient resulted in significant vitamin deficiency presenting as Wernicke encephalopathy. This case highlights the need for detailed, accurate family history and heightened suspicion for malnutrition in young adults who have eating disorders, dysphagia, and unexplained neurological changes.

## Introduction

Malnutrition is commonly present in hospitalized patients and often remains unnoticed until clinically dramatic deterioration has occurred [1]. Elderly populations are at a disproportionately higher risk of malnutrition upon hospital discharge [2]. Malnutrition in young adults is as prevalent and often presents due to underlying severe conditions such as traumatic injuries, surgery, prolonged intensive care unit (ICU) stay, and gastric bypass surgery. In this population, a few rare genetic disorders can also contribute to malnutrition if not identified early. One such disorder is adult-onset myotonic dystrophy type 1 (DM1), which is caused by alterations in the gene that affects skeletal and smooth muscles and is known to cause gastrointestinal dysmotility and malnutrition in young adults [3]. Unlike other common neuromuscular disorders such as amyotrophic lateral sclerosis [4-6] and Duchenne muscular dystrophy [7,8], guidelines on the management of nutritional deficiency in myotonic deficiency are not widely available.

Here, we present the case of a young healthy adult who was admitted with Wernicke encephalopathy secondary to malnutrition caused by undiagnosed DM1. To our knowledge, there are no reported cases of severe malnutrition with underlying undiagnosed DM1 in a young healthy adult.

This article was previously presented as a poster at the 2021 USA Resident and Fellow Scholarship Exposition on June 17, 2021, Mobile, AL, USA.

## Case presentation

History

A 34-year-old female with a history of ovarian cystadenoma resection six weeks prior to admission presented with acutely altered mental status and generalized weakness for two days after undergoing an esophagogastroduodenoscopy (EGD). As the patient was not fully oriented, history was obtained from her next of kin. According to him, the patient had been suffering from intractable nausea and vomiting with an inability to tolerate oral diet and subsequent weight loss for the past three to four months for which she had undergone EGD two days prior to her most recent acute presentation with altered mental status. One day prior to admission, she had become increasingly confused and was unable to walk due to generalized weakness. There was no history of fever, urinary complaints, cough, altered bowel movements, or recent infections. The patient did not smoke, drink alcohol, or use illicit drugs. Family history was significant for an undiagnosed muscular disorder which required her father to ambulate with a wheelchair in the fourth decade of his life.

Physical examination

The patient was tachycardic with a heart rate of 122 beats per minute and hypotensive with a blood pressure of 97/60 mmHg with cool extremities. She was afebrile with a respiratory rate of 16 breaths per minute. Systemic examination was unremarkable. Neurological examination revealed hyporeflexia, nystagmus, decreased strength in lower limbs, and orientation to only person and place.

Lab investigations and management

**Figure 1:**
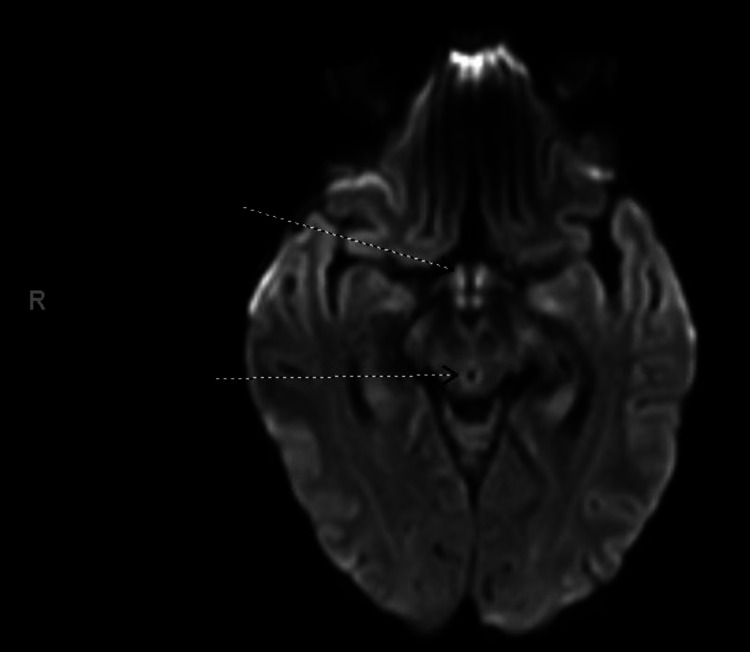
T2-FLAIR hyperintensities in the periaqueductal gray, medial thalamus, and mamillary bodies that were consistent with non-alcoholic Wernicke encephalopathy. FLAIR: fluid-attenuated inversion recovery

At presentation, lab workup showed urinary tract infection secondary to pan-sensitive *Escherichia coli*, for which treatment was started. Eventually, the patient was intubated for airway protection secondary to worsening encephalopathy. Magnetic resonance imaging (MRI) of the brain was performed which showed T2/fluid-attenuated inversion recovery hyperintensity in the periaqueductal gray, medial thalami, and mamillary bodies consistent with Wernicke encephalopathy (Figure 1). Thiamine and other vital micronutrient and vitamin levels were found to be significantly low. This, along with findings of nystagmus and acute onset of confusion, strongly suggested the diagnosis of malnutrition and Wernicke encephalopathy. Thiamine and other micronutrients were aggressively repleted leading to the gradual improvement of the patient’s encephalopathy. However, the cause of the patient’s chronic dysphagia and subsequent malnutrition remained unknown. Given the family history of a possibly undiagnosed muscular disorder, genetic testing was performed on this admission which showed DM1 with 11 trinucleotide repeats of cytosine-thymine-guanine (CTG) in allele 1 and more than 150 in allele 2 of the *myotonic dystrophy protein kinase* (*DMPK*) gene. The patient’s progressive dysphagia was likely a chronic manifestation of myotonic dystrophy that only became more apparent following general anesthesia. Despite improvement in mental status, the patient remained intubated for several weeks due to suboptimal negative inspiratory effort. Nutritional status improved following enteral nutrition. Due to the patient’s continued inability to tolerate an oral diet, a percutaneous esophagogastrostomy (PEG) tube was placed. Eventually, the patient was discharged to a rehabilitation facility with a tracheostomy and PEG tube.

## Discussion

DM1, also known as Steinert’s disease, affects approximately 1 in 8,000 people [9]. It is caused by the mutation of the *DMPK* gene. The repetition of the CTG sequence of this gene present on chromosome 9 affects largely the skeletal and smooth muscles. DM1 is inherited in an autosomal dominant pattern and exhibits certain non-Mendelian characteristics such as somatic mosaicism, variable penetrance, and anticipation. This pattern of transmission is responsible for a large variety of variable signs and symptoms and effects on multiple organ systems which often makes the correct diagnosis elusive.

Adult-onset DM1, often diagnosed in the third or fourth decade of life, characteristically presents with myotonia muscle weakness and early cataracts. However, these features are not always present in a majority of cases, and other organ systems may be affected [10]. It may adversely affect the respiratory system (via respiratory muscle weakness or central mediation) making respiratory failure the leading cause of death [11]. Cardiac arrhythmias, heart blocks, and cardiomyopathies can also occur and are the second leading cause of death in patients with DM1. Gastrointestinal (GI) disturbances are also common in DM1 and can be disabling in up to 25% of patients [12], as in the case of our patient. These disturbances commonly include dysphagia, bloating, and abdominal fullness. Lower GI symptoms such as diarrhea, constipation, and fecal incontinence can also be present. Of note, DM1 symptoms are aggravated after receiving general anesthesia [13]. Our patient’s symptoms of nausea, vomiting, and dysphagia worsened after she received general anesthesia.

The diagnosis of myotonic dystrophy begins with a thorough history and physical examination. It is imperative to obtain a thorough history. Our patient had a family history of “undefined muscular disorder” in the father that had made him wheelchair-bound in the fourth decade of life which prompted us to evaluate her for muscular disorders. Another clue was the acute worsening of her symptoms after exposure to general anesthesia.

Moreover, at presentation, our patient had neurological signs and symptoms consistent with Wernicke encephalopathy secondary to severe malnutrition. The leading cause of Wernicke encephalopathy in the United States continues to be chronic alcohol use [14], and it is uncommon to have severe malnutrition presenting as acute WE in the absence of alcohol use. Hence, it is important to not only evaluate thiamine levels but also other vitamin and micronutrient levels. There is a lack of evidence-based data and guidelines on the management of malnutrition in patients with DM1. We aggressively repleted her nutritional deficiencies through enteral feeding. Tolerance of enteral feeds improved with the addition of metoclopramide as there is evidence of promotility agent efficacy in DM1 [10,15]. Eventually, the patient required nutrition through a PEG tube.

## Conclusions

This case highlights the importance of recognizing the signs of malnutrition in a young otherwise healthy adult who presents with dysphagia or an eating disorder. It is important to investigate the cause of malnutrition and dysphagia by obtaining a detailed history coupled with a thorough physical examination. In our patient, we managed to uncover her longstanding missed diagnosis of DM1 when her malnutrition caused Wernicke encephalopathy. This case also stresses the need to develop guidelines to manage malnutrition in patients with DM1.
